# Advantages of the Silkworm As an Animal Model for Developing Novel Antimicrobial Agents

**DOI:** 10.3389/fmicb.2017.00373

**Published:** 2017-03-07

**Authors:** Suresh Panthee, Atmika Paudel, Hiroshi Hamamoto, Kazuhisa Sekimizu

**Affiliations:** Institute of Medical Mycology, Teikyo UniversityTokyo, Japan

**Keywords:** silkworm model, pharmacokinetics, therapeutic activity, lysocin E, novel antibiotics

## Abstract

The demand for novel antibiotics to combat the global spread of multi drug-resistant pathogens continues to grow. Pathogenic bacteria and fungi that cause fatal human infections can also kill silkworms and the infected silkworms can be cured by the same antibiotics used to treat infections in the clinic. As an invertebrate model, silkworm model is characterized by its convenience, low cost, no ethical issues. The presence of conserved immune response and similar pharmacokinetics compared to mammals make silkworm infection model suitable to examine the therapeutic effectiveness of antimicrobial agents. Based on this, we utilized silkworm bacterial infection model to screen the therapeutic effectiveness of various microbial culture broths and successfully identified a therapeutically effective novel antibiotic, lysocin E, which has a novel mode of action of binding to menaquinone, thus leading to membrane damage and bactericidal activity. The similar approach to screen potential antibiotics resulted in the identification of other therapeutically effective novel antibiotics, such as nosokomycin and ASP2397 (VL-2397). In this regard, we propose that the silkworm antibiotic screening model is very effective for identifying novel antibiotics. In this review, we summarize the advantages of the silkworm model and propose that the utilization of silkworm infection model will facilitate the discovery of novel therapeutically effective antimicrobial agents.

## Introduction

The conventional approach of antibiotic discovery includes purification from culture supernatants of soil bacteria by monitoring *in vitro* antimicrobial activity. This approach makes it difficult to identify novel antibiotics, however, due to the frequent isolation of overlapping chemical entities. In addition, only a small fraction of the antibiotics isolated using this approach exert therapeutic activity in animal models, which further limits the discovery of the therapeutically effective antibiotic. The lack of therapeutic activity by most of the compounds is due to their toxicity and poor pharmacokinetic properties. Thus, the conventional approach of antibiotic screening clearly requires remodeling. To overcome the problems associated with conventional screening methods, we used the silkworm as an animal model to evaluate the therapeutic effects of candidate samples. In this review, we discuss the advantages of the silkworm model for antimicrobial drug development.

## Advantages of the silkworm as an animal model

Silkworms, the larvae of the domesticated moth *Bombyx mori*, have been used for silk production for more than 5,000 years. The silk industry originally started in China, distributed toward several parts of Asia and the West, and has contributed greatly to the economic development of countries along the Silk Road. While *B. mori* continues to be a major player in sericulture throughout the world, it has also gained importance for biotechnology as a bioreactor for the production of recombinant proteins and silk-based biomaterials (Altman et al., 2003; Kato et al., 2010). From an anatomic point of view, *B. mori* harbors most of the organs and systems present in mammals, leading scientists to use *B. mori* as an excellent model organism to elucidate various processes in life sciences, which has been facilitated by the availability of its complete genome sequence and the development of technologies for genetic manipulation. As an animal model, *B. mori* has clear advantages over other organisms (Table 1).

**Table 1:** **Invertebrate animal models**.

**Species**	**Size (mm)**	**Behavior**	**Special equipment for organ isolation**	**Chance of biohazard**	**Special handling technique for administration**	**Route of administration/accuracy of administered dosage**	**Studies on immunity**
Fruitfly (*Drosophila melanogaster*)	1–3	Adult: fly	Required	High	Required	Oral, injection to dorsal surface, not accurate	Extensive
Grasshopper (*Locusta migratoria*)	60–80	Jump	Not required	High	Required	–	Little
Honey bee (*Apis mellifera*)	15–17	Adult: fly	Not required	High	Required	–	Extensive
Waxmoth (*Galleria mellonella*)	30–40	Larvae: faster than silkworm	Not required	Higher than silkworm, less than others	Not required	Oral, topical, injection to ventral surface/accurate in case of injection	Extensive
		Adult: fly					
Silkworm (*Bombyx mori*)	50–60	Larvae: slow	Not required	Low	Not required	Oral, injection to dorsal surface: intra hemolymph, intra midgut/accurate in the case of injection	Extensive
		Adult: don't fly					

### Ethical issues

Over the last several years, animal welfare concerns have forced scientists to reduce the number of vertebrates, especially mammals, used as experimental animal models, and alternative animal models that do not require approval by the ethics committee have been sought. Given that silkworms have been used in the silk industry for centuries and their application for research does not require ethical clearance, their use as experimental animal models is both less complicated and less costly compared to vertebrates.

### Rearing system

The utilization of silkworms in the silk industry has facilitated its domestication. The proper methods of feeding and maintaining silkworms are well-established. The development of artificial diet in the 1960s replaced the seasonally available mulberry leaves and allowed year-round utilization of silkworms. The feeding of artificial diet not only facilitated breeding and rearing but also contributed to a uniform quality of silkworms. Uniformity in quality is important for robust and reproducible results in studies performed using model animals. The widespread distribution of the silk industry makes it easy to obtain fertilized eggs, thus dramatically reducing the time and labor required to care for silkworms. As fifth-instar day 2 larvae are utilized for infection assays, the total silkworm rearing time required from hatching of the eggs to using the larvae for infection assays is <3 weeks, which is far shorter than the time required for mammals. Utilization of aseptic procedures during rearing allows the generation of germ-free larvae. Furthermore, larvae molt four times; thus making it easy to distinguish each instar stage and reducing individual genetic differences.

### Cost issue

A silkworm rearing room generally includes an incubator and a safety cabinet. Thus, silkworm rearing does not require large and expensive equipment, making it much less expensive than other animal models. Furthermore, in contrast to mammalian models, large numbers of larvae can be reared in a single cage, which significantly reduces the cost of maintaining silkworms. Based on our experience, the cost of using silkworms is 1% that of the cost of utilizing mice.

### Handling

**Figure 1:**
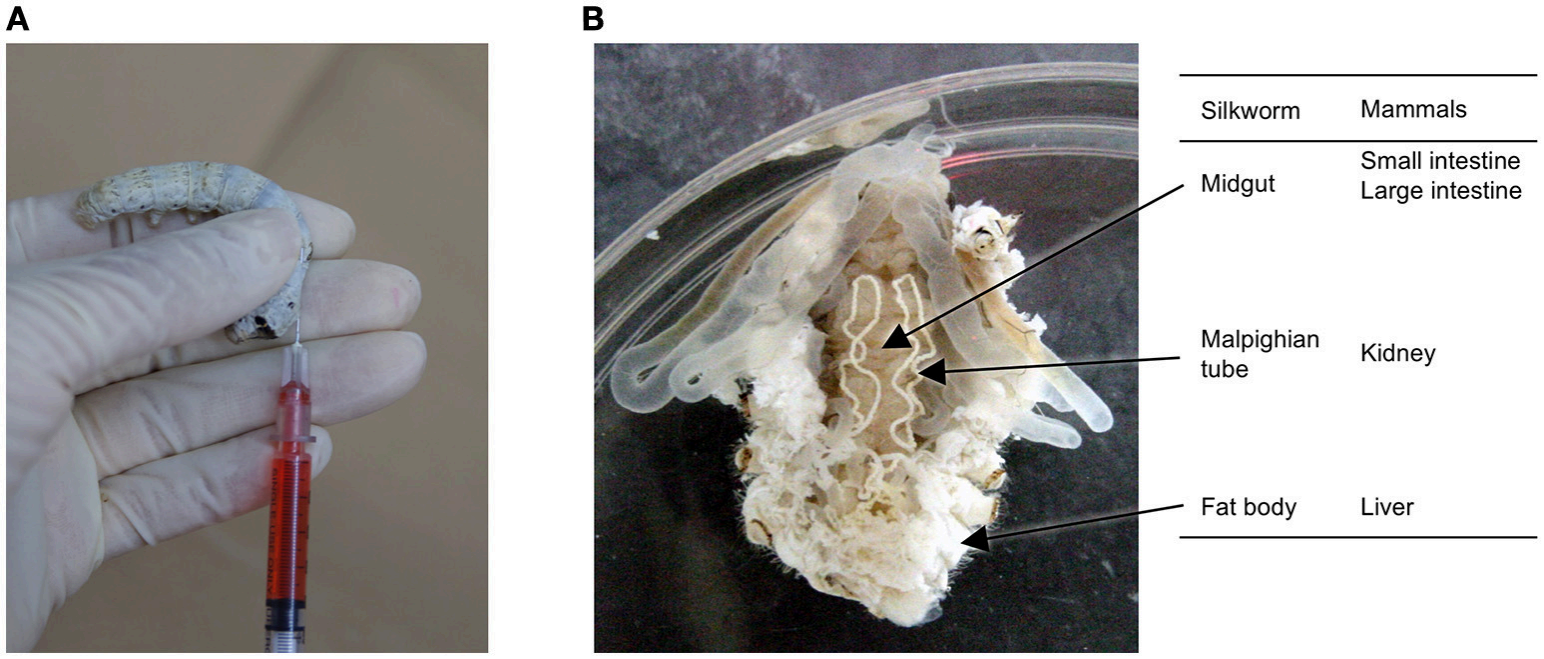
**Silkworm and its organs. (A)** Manual injection of a sample into the silkworm hemolymph using a 1-ml disposable syringe, and **(B)** comparison of silkworm organs involved in drug absorption, metabolism, and elimination between silkworms and mammals.

In general, experiments utilizing silkworms require less time than those utilizing mammalian models. Further, silkworm larvae have a large enough body size for easy handling by human hands, and samples can be injected with the same syringe type used for medical purposes in humans (Figure 1A). Samples can be injected into the hemolymph, which corresponds to intravenous injection in mammals, and the midgut, corresponding to oral administration. Moreover, silkworm organs such as the midgut can be isolated for experimental use. Silkworms are easy to work with; they do not have sharp horns/hair, teeth, or claws that can sting, and do not bite. Moreover, the adult moths cannot fly and the movement of silkworm larvae is slow and weak, making them easy to handle for injecting samples. This weak and slow movement also makes it difficult for silkworms to escape from their cages, allowing us to perform infection assays for level 2 pathogens. As silkworms have been very highly domesticated through the long history of the silk industry, they cannot survive or reproduce in a natural environment. This further adds to the safety of using silkworms for injection of pathogenic organisms and reduces the biohazard potential.

### Gene editing

Attempts to decode the *B. mori* genome were performed in 2004 and 2008 (Xia et al., 2004; The International Silkworm Genome Consortium, 2008). The availability of the genome sequences facilitated the development of basic genetic and molecular genetic tools and markers. Over the last decade, the development of genetic technologies for *B. mori* has greatly advanced. Genetic modification may be achieved by transposon-based technology, such as transgene integration or expression; RNA interference-based gene silencing; gene- and enhancer-trap methods and genome editing technology utilizing zinc finger nucleases; transcription activator-like effector nucleases (TALENs); and CRISPR/Cas9 (Xu and O'Brochta, 2015). Furthermore, Dr. Sezutsu's group in the National Agriculture and Food Research Organization, Japan, recently succeeded in utilizing these various techniques to edit the silkworm genome (Inagaki et al., 2015; Sakurai et al., 2015; Takasu et al., 2016). With these recent developments, the silkworm now has a sophisticated genetic modification system and can thus be used to establish disease models and humanized models to screen candidate compounds and analyze physiologic processes.

### Common pharmacokinetics between silkworm and mammals

Silkworm organs, such as the gut, fat body, and malpighian tubule, correspond to the intestine, liver, and kidney, respectively, in mammals, and are involved in the metabolism and excretion of external compounds (Figure 1B). Pharmacokinetic parameters are very important from the view of therapeutic activity. Even compounds that exhibit good activity *in vitro* will not be effective *in vivo* if they have poor pharmacokinetic parameters. We demonstrated that the half-lives of model compounds in silkworms are similar to those in mammals (Hamamoto et al., 2009). We also found that a model compound is metabolized in silkworms by the cytochrome P450 enzyme, follows the metabolic pathway via the conjugation reaction, and exhibits the similar pharmacokinetics as in mammals (Hamamoto et al., 2009). We also demonstrated that the general non-specific transport of molecules through paracellular routes is comparable between the mammalian intestine and the silkworm midgut (Hamamoto et al., 2004, 2005). In addition, the doses of cytotoxic chemicals that are lethal in 50% of the animals (LD_50_) are similar between silkworms and mammals, indicating that the toxicity of compounds listed in the OECD guidelines can be evaluated using silkworms (Usui et al., 2016). Based on this, we have used silkworms to optimize antimicrobial agents for less acute toxicity and succeeded to increase the median lethal dose (LD_50_) from 100 to 230 μg/g larvae (Paudel et al., 2013).

### Host–pathogen interaction and immune response in silkworm

Pathogens respond and adapt to the signals appearing in the host environment to successfully infect the host and in most cases, the host's response is critical to determine the degree of pathogenicity. An ideal animal model should provide a response similar to that of humans, during infections. Although insects like silkworm lack acquired immunity, innate immunity is widely conserved among mammals and insects (Hoffmann and Reichhart, 2002). Immune response by silkworms includes humoral response such as: production of various proteins like phenoloxidase, antimicrobial peptides, lysozymes, lectins, serine proteases, etc.; and cellular response such as hemocyte-mediated phagocytosis, encapsulation, nodule formation, etc. We previously reported the activation of innate immunity in silkworm during pathogen invasion and found that a cytokine-like paralytic peptide induced humoral and cellular responses and played an important role in silkworm immunity. Further, we found that the paralytic peptide promoted the engulfment of bacteria and induced nitric oxide (NO) production, that is required for both p38 and MAPK activation (Ishii et al., 2013, 2015a).

All the features of silkworms mentioned above suggest that they are a suitable alternative evaluation system for determining the therapeutic effectiveness of candidate drugs. In addition, the availability of a large number of silkworms with low cost and no ethical issues allows us to screen novel antibiotics for their therapeutic effectiveness.

## Advantage of screening novel antibiotics using the silkworm infection model

We have previously shown that pathogenic bacteria and fungi that cause fatal infections in human beings such as methicillin sensitive and resistant *Staphylococcus aureus*; *Pseudomonas aeruginosa* can also kill silkworms. Furthermore, these infections were cured with the same antibiotics used clinically to cure these infections (Kaito et al., 2002; Hamamoto et al., 2004). Since then, silkworms have been utilized to study pathogenic bacterial toxins (Hossain et al., 2006); evaluate the target specificity of antibacterial agents (Kurokawa et al., 2009); identify novel *S. aureus* virulence genes (Kaito et al., 2005; Miyazaki et al., 2012); and identify novel probiotic bacteria that promote survival during *P. aeruginosa* infection (Nishida et al., 2016). Additionally, infection model of multiple pathogenic microorganisms *Stenotrophomonas maltophilia* (Hamamoto et al., 2004); *Vibrio vulnificus* (Yamamoto et al., 2016); *Vibrio cholera* (Kaito et al., 2002); *Candida tropicalis* (Hamamoto et al., 2004); *Candida albicans* (Hamamoto et al., 2004); *Aspergillus fumigatus* (Nakamura et al., 2017a); *Cryptococcus neoformans* (Matsumoto et al., 2012); and laboratory generated vancomycin-resistant *S. aureus* VR7 (Tabuchi et al., 2017) have been developed and the success of the antimicrobial to rescue the silkworms from the effect of pathogens have been reported (Table 2). These findings suggest that silkworm infection models can be utilized to mimic the infections caused by various bacterial and fungal pathogens.

**Table 2:** **Silkworm infection model and therapeutic effect of antimicrobial agents**.

**Infection model**	**Treated with**	**ED_50_ (μg/larvae)**	**References**
Methicillin-susceptible *S. aureus*	Kanamycin	3	Hamamoto et al., 2004
	Arbekacin	4	
	Teicoplanin	0.3	
	Vancomycin	0.3	
	Tetracycline	0.4	
	Minocycline	3.9	
	Chloramphenicol	7	
	Flomoxef	0.2	
	Linezolid	9	
Methicillin-resistant *S. aureus*	Vancomycin	<6.5	Kaito et al., 2002
Vancomycin-resistant *S. aureus* VR7	Vancomycin-ceftriaxone	ND	Tabuchi et al., 2017
*Stenotrophomonas maltophilia*	Minocycline	7.8	Hamamoto et al., 2004
	Sulfamethoxazole-trimethoprim	57	
	Imipenem-cilastatin	50	
*Candida tropicalis*	Amphotericin B	1.8	Hamamoto et al., 2004
	Fluconazole	1.8	
*Candida albicans*	Amphotericin B	4.1	Hamamoto et al., 2004
	Fluconazole	1.8	
*Cryptococcus neoformans*	Amphotericin B	14	Matsumoto et al., 2012
	Flucytosine	6	
	Fluconazole	2	
	Ketokonazole	19	

**Figure 2:**
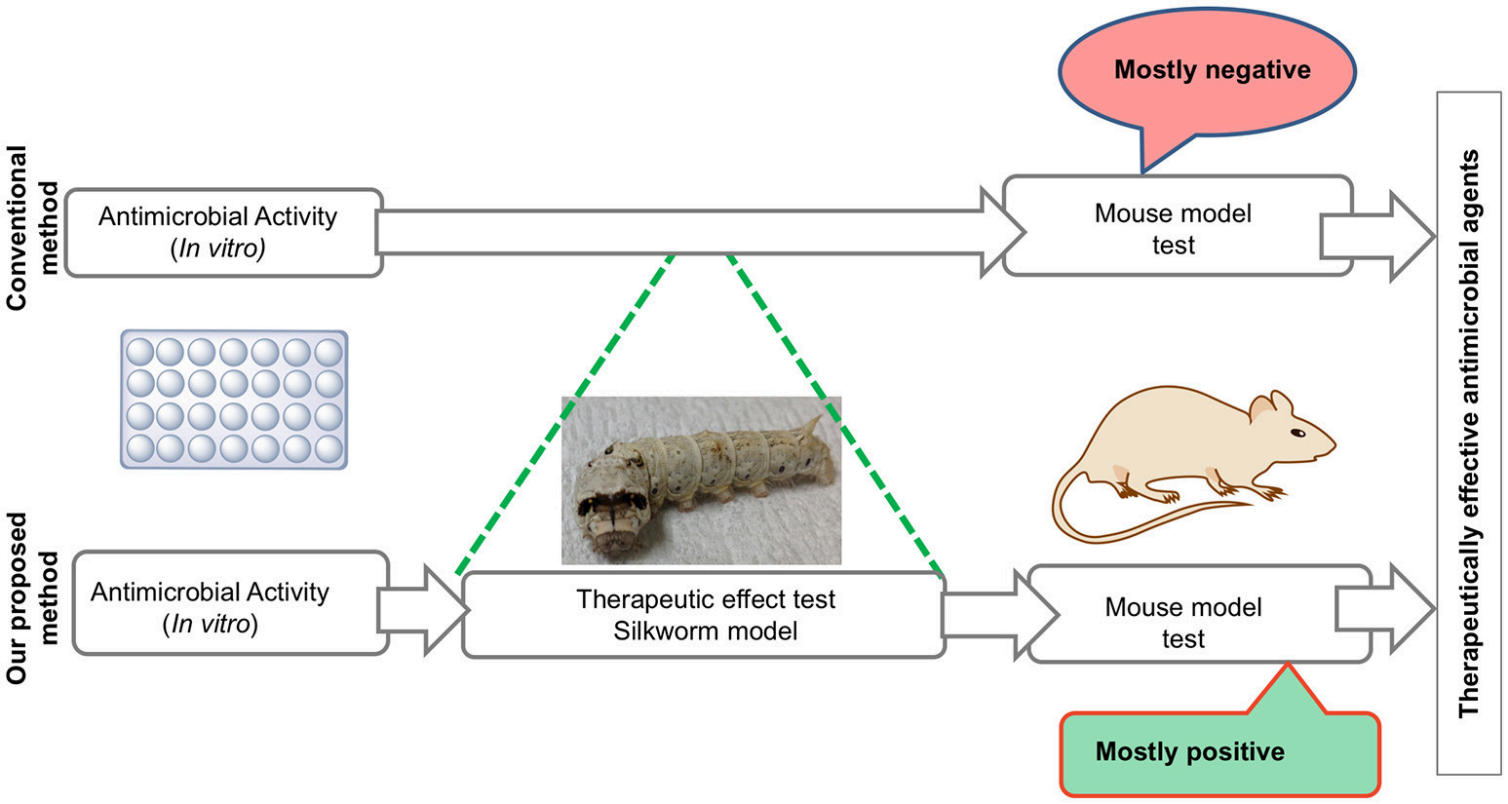
**Utilization of silkworms to screen therapeutically active antimicrobial agents**.

We also demonstrated that the dose required to cure 50% of fatal infections (ED_50_) in silkworms is similar to that in mice, suggesting that the pharmacokinetics of these antibiotics are similar between silkworms and mammals (Hamamoto et al., 2004). This shared common feature in the pharmacokinetic parameters of clinically applied antibiotics between mammals and silkworms led us to utilize the silkworm infection model to screen for antimicrobial agents. Given that most of the antibiotics currently used clinically were derived from natural products isolated from microorganisms and these microorganisms serve as a repertoire of novel antibiotics, the utilization of silkworms has a further advantage—the possibility of injecting a crude extract. The injection of a crude extract from natural products, such as plant extracts and supernatant of soil bacteria, is prohibited in mammals by the experimental guidelines for animal welfare due to the lack of a rational basis for the experiment. Soil bacteria can produce several anti-bacterial agents, which are often effective only in *in vitro* conditions and do not show therapeutic efficacy. Purification based on *in vitro* activity alone may miss to identify therapeutically effective compounds. Furthermore, our previous experience of selecting the hits from a chemical library using minimum inhibitory concentrations (MIC) as an indicator showed that these compounds had no therapeutic activity (Paudel et al., 2012, 2013). Therefore, we chose to evaluate the therapeutic activity using the silkworm infection model to purify therapeutically effective compounds. By doing so, we can easily eliminate the compounds that are only effective *in vitro* and focus on therapeutically effective compounds, even when their production level is lower than that of compounds that are only effective *in vitro*, which reduces time, effort, resources, and cost to obtain a therapeutically effective compound (Figure 2). These features of the silkworm infection model facilitate the identification of novel, therapeutically effective antibiotics from natural products.

## Development of novel antimicrobial agents using the silkworm infection model

We developed the silkworm infection model by evaluating the effects of various pathogenic bacteria and fungi. Further, antibiotics and antifungal agents clinically used against those bacteria and fungi effectively cured infections in silkworms. By utilizing the silkworm infection model, we and collaborators successfully identified novel antimicrobial agents—lysocin E (Hamamoto et al., 2015), nosokomycins (Uchida et al., 2010), and ASP2397 (Nakamura et al., 2017a). The details of the discovery of lysocin E and ASP2397 are presented in this review.

### Identification of novel antibiotics lysocin E from soil bacteria

**Figure 3:**
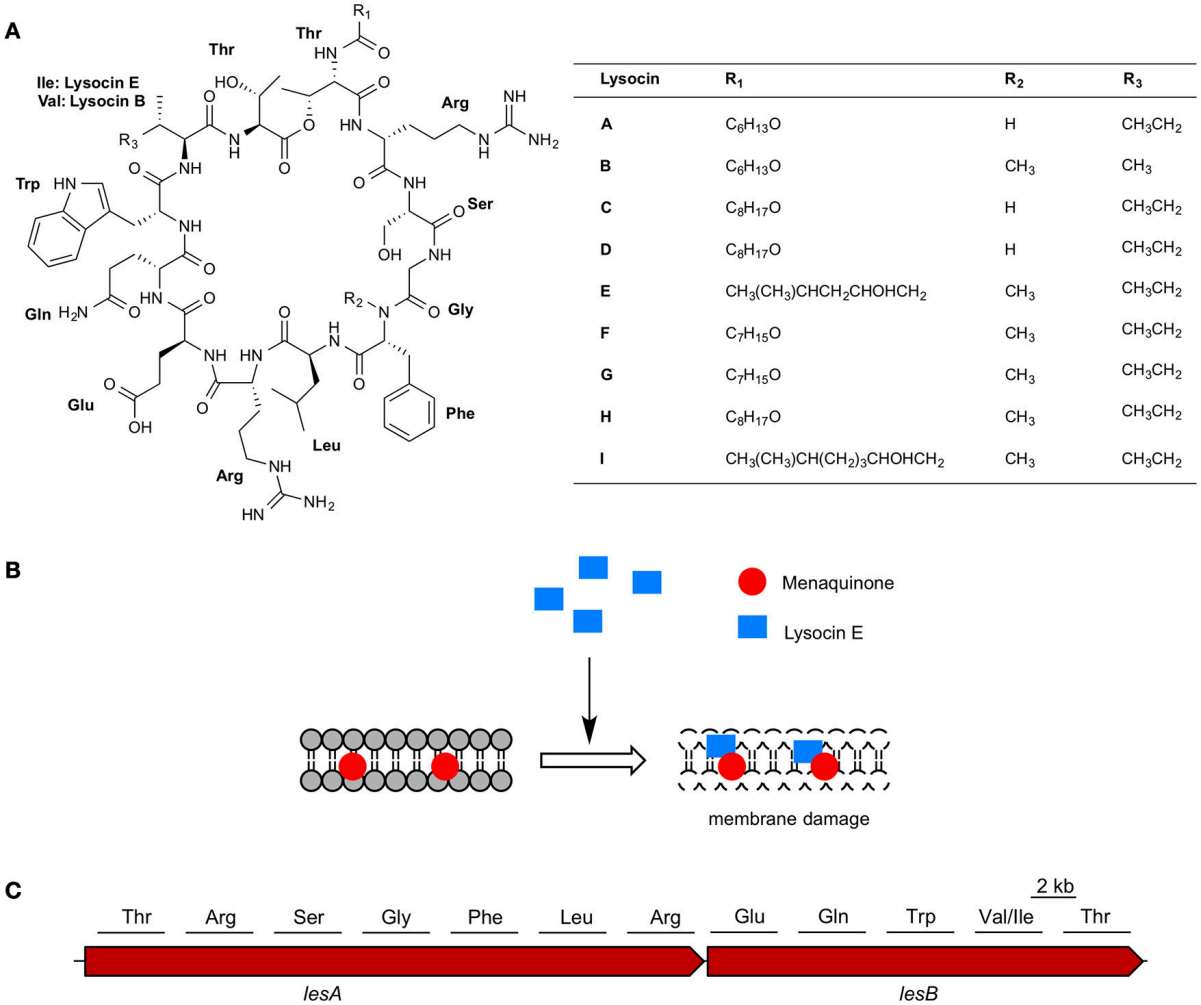
**Lysocins, mode of action, and biosynthesis. (A)** Chemical structure of lysocins A–I and **(B)** schematic representation of membrane damage by lysocin E **(C)** non-ribosomal peptide synthetases involved in lysocin biosynthesis and amino acids activated by the 12 modules in LesA and LesB.

We collected soil samples from various places within Japan and isolated bacteria from these samples. We screened the supernatants of the soil bacteria for *in vitro* antimicrobial activity against methicillin-resistant *Staphylococcus aureus* as a primary screening. Of the 14,651 supernatants, 2,794 (19%) had *in vitro* inhibitory activity against *S. aureus*. Surprisingly, only 23 of the 2,794 culture supernatants had therapeutic activity in the silkworm infection model. This low hit rate for the therapeutic effectiveness of the culture supernatants indicates that the silkworm infection model is highly effective for excluding antibiotics that do not have therapeutic effectiveness. We purified antibiotics from the culture supernatant of a *Lysobacter*, which has recently attracted high interest due to their capacity to produce antibiotics and other natural products (Panthee et al., 2016). Purification based on the therapeutic activity observed in silkworms infected with *S. aureus* from the culture broth of *Lysobacter* sp. RH2180-5 led to the identification of a therapeutically active novel antibiotic, lysocin E (Hamamoto et al., 2015) (Table 3, Figure 3A). The detailed purification process showed that the *in vitro* activity was higher for the partially purified butanol extract than purified lysocin E. This indicated that the culture broth contained both therapeutically active and inactive antimicrobial components and our success in finding a therapeutically active antibiotic relied on utilization of the silkworm screening system.

**Table 3:** **Purification of lysocin E from the culture supernatant of ***Lysobacter*** RH2180-5**.

**Fractions**	**MIC (μg/ml)**	**ED_50_ (μg/g)**
Acetone extract	25	90
Butanol extract	0.6	4
Water precipitation	N.D.	1.8
ODS column chromatography	N.D.	0.5
Lysocin E	5	0.3

*ND, not determined*.

Lysocin E is a cyclic peptide that exhibits antimicrobial activity against various Staphylococci, *Bacillus subtilis, Bacillus cereus*, and *Listeria monocytogenes* with MICs ranging from 1 to 4 μg/ml (Table 4). Based on the genetic and biochemical analysis, we identified menaquinone, a membrane molecule important for the bacterial electron transport chain, as the lysocin E target (Figure 3B). Lysocin E is the first antibiotic discovered that targets menaquinone (Hamamoto et al., 2015; Paudel et al., 2016). Lysocin E also has potent therapeutic activity in a mouse infection model of methicillin-resistant *S. aureus* (ED_50_: 0.5 mg/kg). Lysocin E did not exhibit acute toxicity in mice at a dose up to 400 mg/kg. The chemical structure of lysocin E comprises 12 amino acids arranged linearly by two large multimodular non-ribosomal peptide synthetases named lysocin E synthetase (LesA and LesB) that have a total of 12 modules and 43 domains in a 1.7-MDa core peptide (Figure 3C) (Panthee et al., 2017). The potent therapeutic activity and low toxicity of lysocin E in animal infection models suggest its high potential for clinical application and the identification of its gene cluster opened avenues for further derivatization of the structure with enhanced activity.

**Table 4:** **MIC of lysocin E against various microorganisms (Hamamoto et al., 2015)**.

**Microorganisms**	**MIC (μg/ml)**
Methicillin-susceptible *S. aureus*	1–4
Methicillin-resistant *S. aureus*	4
Vancomycin-resistant S. aureus^*^	8
*Bacillus* spp.	2
*Listeria monocytogenes*	1
*Mycobacterium* spp.	8
*Serratia marcescens*	>100
*Pseudomonas aeruginosa*	>100
*Candida* spp.	>100
*Cryptococcus neoformans*	>100
*Escherichia coli* W3110	>100
*Streptococcus* spp.	>128

* *Laboratory generated vancomycin-resistant strain (Ishii et al., 2015b)*.

### Identification of a novel antifungal compound, ASP2397 (VL-2397)

**Figure 4:**
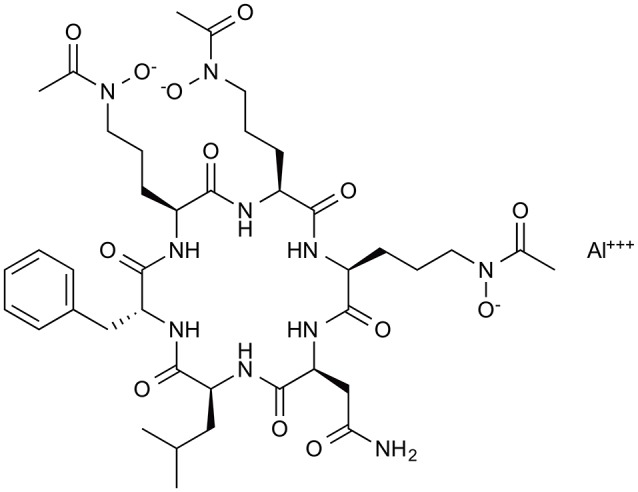
**Chemical structure of the novel antifungal compound ASP2397 (VL-2397)**. ASP2397, purified based on *in-vivo* therapeutic effect in silkworm infection model.

To identify antifungal compounds, the clinical fungal isolate *A. fumigatus* FP1305 was used as a test strain. Astellas group screened culture broths of 310 fungal strains for *in vitro* activity against *A. fumigatus* FP1305, followed by testing the ability of the broths to cure silkworms infected with FP1305. The culture broth of *Acremonium persicinum* MF-347833 had therapeutic activity in the silkworm infection model. To purify the antifungal compound, they first attempted to purify the activity based on the *in-vitro* antifungal activity, which failed to identify some of the therapeutically active fractions. This finding led to the speculation that the culture broth of *A. persicinum* MF-347833 contained a mixture of antifungal compounds with therapeutic activity or without therapeutic activity. They next utilized the silkworm infection assay-guided purification method to identify therapeutically active antifungal compounds and successfully identified ASP2397 (Nakamura et al., 2017a,b) (Figure 4). ASP2397 has an MIC of 0.2 μg/ml against *A. fumigatus* FP1305 and is also effective against azole-resistant *A. fumigatus* (Arendrup et al., 2016), displays therapeutic activity in mice infected with *A. fumigatus*, and has no cytotoxicity toward mammalian cells at a concentration up to 50 μg/ml, indicating its potential as a therapeutic agent. The planar structure of ASP2397 indicates that it is a metal ion chelator that harbors aluminum, indicating the importance of metal chelation for its therapeutic activity. The fact that ASP2397 was not identified by the conventional approach highlights the utility of the silkworm model for identifying therapeutically active drug molecules.

## Conclusion

In summary, we used silkworms as a primary screening system that has several advantages, including an established rearing system, cost effectiveness, reproducible and robust application, no ethical issues, and conserved metabolic pathways with mammals. We established silkworm infection models of various bacteria and fungi and found that silkworm infection model facilitates the discovery of therapeutically effective compounds. Furthermore, we utilized the silkworm infection model to purify compounds from natural sources and identified the antimicrobial agents with therapeutic activity that would otherwise go unidentified. As an example, ASP2397 was identified from *Acremonium persicinum* MF-347833 using silkworms, but was not identified by the purification approach based on *in vitro* activity. Similarly, the purification of lysocin E from a crude extract that had more potent *in vitro* activity than lysocin E itself is an example of how the silkworm screening system avoids some of the restrictions placed on mammal-based screening systems. Thus, the silkworm model is suitable for developing novel therapeutically effective antibiotics from natural products.

## Author contributions

SP wrote the manuscript, AP revised the manuscript, HH determined the outline, and KS critically revised the manuscript and approved the submission.

### Conflict of interest statement

KS is a consultant for Genome Pharmaceutical Institute, Co. Ltd. The other authors declare that the research was conducted in the absence of any commercial or financial relationships that could be construed as a potential conflict of interest.
